# Hemodynamic and recirculation performance of dual lumen cannulas for venovenous extracorporeal membrane oxygenation

**DOI:** 10.1038/s41598-023-34655-1

**Published:** 2023-05-08

**Authors:** Louis P. Parker, Anders Svensson Marcial, Torkel B. Brismar, Lars Mikael Broman, Lisa Prahl Wittberg

**Affiliations:** 1 FLOW, Department of Engineering Mechanics, Royal Institute of Technology, KTH, Osquars Backe 18, SE-100 44 Stockholm, Sweden; 2 Division of Medical Imaging and Technology, Department of Clinical Science, Intervention and Technology at Karolinska Institutet, Stockholm, Sweden; 3 Department of Radiology, Karolinska University Hospital and Karolinska Institutet, Stockholm, Sweden; 4 ECMO Centre Karolinska, Pediatric Perioperative Medicine and Intensive Care, Karolinska University Hospital, Stockholm, Sweden; 5 Department of Physiology and Pharmacology, Karolinska Institutet, Stockholm, Sweden

**Keywords:** Respiratory distress syndrome, Fluid dynamics, Computational models

## Abstract

Venovenous extracorporeal membrane oxygenation (ECMO) can be performed with two single lumen cannulas (SLCs) or one dual-lumen cannula (DLC) where low recirculation fraction (${R}_{f}$) is a key performance criterion. DLCs are widely believed to have lower ${R}_{f}$, though these have not been directly compared. Similarly, correct positioning is considered critical although its impact is unclear. We aimed to compare two common bi-caval DLC designs and quantify ${\mathrm{R}}_{\mathrm{f}}$ in several positions. Two different commercially available DLCs were sectioned, measured, reconstructed, scaled to 27Fr and simulated in our previously published patient-averaged computational model of the right atrium (RA) and venae cavae at 2–6 L/min. One DLC was then used to simulate ± 30° and ± 60° rotation and ± 4 cm insertion depth. Both designs had low ${R}_{f}$ (< 7%) and similar SVC/IVC drainage fractions and pressure drops. Both cannula reinfusion ports created a high-velocity jet and high shear stresses in the cannula (> 413 Pa) and RA (> 52 Pa) even at low flow rates. Caval pressures were abnormally high (16.2–23.9 mmHg) at low flow rates. Rotation did not significantly impact ${R}_{f}$. Short insertion depth increased ${R}_{f}$ (> 31%) for all flow rates whilst long insertion only increased ${R}_{f}$ at 6 L/min (24%). Our results show that DLCs have lower ${R}_{f}$ compared to SLCs at moderate-high flow rates (> 4 L/min), but high shear stresses. Obstruction from DLCs increases caval pressures at low flow rates, a potential reason for increased intracranial hemorrhages. Cannula rotation does not impact ${R}_{f}$ though correct insertion depth is critical.

## Introduction

Historically, venovenous extracorporeal membrane oxygenation (VV ECMO) has been performed with dedicated drainage and return cannulas. The concept of the dual-lumen cannula (DLC), which combines these two functions into a single device, emerged in the mid-80’s^1^. The single cannulation site makes cannulation safer, faster and allows for greater patient mobility.

Recirculation fraction ($${R}_{f})$$ is the proportion of oxygenated return blood which is drained from the native circulation before passing through the tricuspid valve thus not contributing to patient oxygenation. To achieve a low $${R}_{f}$$ is a key performance criterion for any VV ECMO cannulation strategy. DLCs are widely believed to have reduced $${R}_{f}$$ compared to single lumen cannulas (SLCs). Whilst the efficacy of DLCs for adult VV ECMO has been demonstrated in several studies^2–4^, $${R}_{f}$$ has not been directly reported. Thus, the literature lacks sufficient evidence to conclusively support superior performance^5^.

Ultrasound dilution technique can be used to measure ${R}_{f}$^6^, for the most part, high pre-membrane lung saturation ${(S}_{Pre}{O}_{2})$ is the typical clinical indicator of cannula malposition. Indications are that ${R}_{f}$ is sensitive to cannula positioning^7^. Data on the precise relationship between DLC position and ${R}_{f}$ in adult ECMO is absent.

We have developed a computational fluid dynamics (CFD) model of the right atrium (RA) and venae cavae^8^, tested its sensitivities^9^, and validated it against clinical ${R}_{f}$ data^10^. The model serves as a platform to investigate the impact of VV ECMO cannulation variables on performance. In this study we applied our CFD model to compare the hemodynamics of two common DLC designs, assessing the relationship between cannula rotation and insertion depth with performance. Cannula performance in each DLC was assessed by ${R}_{f}$ and the exposure of blood to high shear stresses, for a range of ECMO flow rates.

## Methods

**Figure 1:**
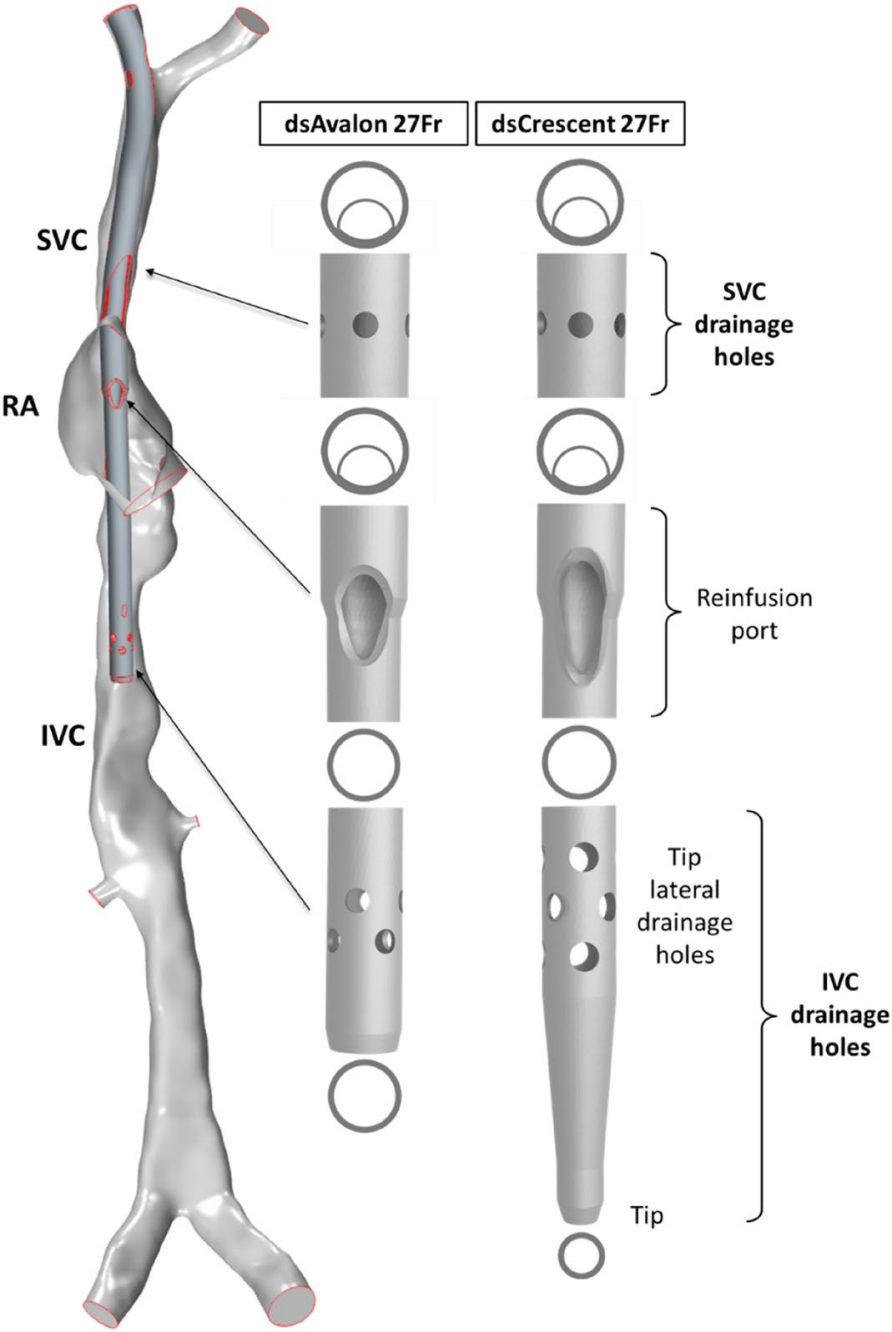
The patient-averaged right atrium, superior (SVC) and inferior vena cava (IVC) model with the dsAvalon inserted in the baseline position (left). Comparison of the main design features of the dsAvalon and dsCrescent DLCs (right).

All methods were performed in accordance with the relevant guidelines and regulations and ethical approval was obtained from the Swedish Ethical Review Authority. A rigid-wall patient-averaged model of the adult RA and venae cavae reported previously was used to model venous flow^8^. This model was based on four healthy volunteers (3 female, 1 male) with mean age = 58.3 ± 3.5yrs, weight = 77.0 ± 11.9 kg and height = 173.0 ± 2.8 cm. Averaging of the geometry was achieved by quantifying minimum/maximum diameters and length for each venous branch as well as the dimensions of the right atrium. Using these, individual vein segments were selected, scaled and merged to create a model that best reflected the group mean of these measurements, as previously described^8^. A constant total venous return of 6 L/min was applied with 35% entering the RA from the superior vena cava (SVC) and 65% from the inferior vena cava (IVC)^11,12^. These inflows were then distributed amongst the venous branches by inlet area. Two commonly used DLC designs, the Maquet Avalon Elite® (Getinge, Rastatt, Germany) and MC3 Crescent® (Medtronic International Trading Sàrl, Tolochenaz, Switzerland) were sectioned manually with a blade, measured with vernier calipers, and reconstructed into accurate 3D CAD geometries. Following the same methodology, as previously used by us with SLCs^10^, we positioned 3D reconstructions of each device in the patient-averaged model following venous centrelines, also modelling them as rigid-wall. A 27Fr cannula size was selected for the patient-averaged model mimicking a reasonable clinical choice of cannula size. Using available physical cannula samples, a 30Fr Crescent and 31Fr Avalon cannula were both downscaled to 27Fr. In downscaling the cannulas, the original wall thicknesses and lateral drainage hole diameters were maintained whilst lumen diameters and the reinfusion port were scaled according to Fr size. Cannula lengths were unchanged, as is the case for Avalon and Crescent cannulas in this size range. To delineate that these are not direct reconstructions of the off-the-shelf products but downscaled (ds) representations, the DLCs are thereafter referred to as dsCrescent and dsAvalon throughout. Comparison of the design features of each device is shown in Fig. 1. Comparisons to SLCs reference our previous publication^10^ where we inserted a 25Fr Maquet HLS Multistage drainage cannula and 19Fr Medtronic Bio-Medicus return cannula in atrio-femoral (drainage in the SVC) and femoro-atrial (drainage in the IVC) configurations, using the same patient-averaged model.

### CFD model

The CFD model follows the same setup used previously^8–10^. Briefly, it is a large Eddy simulation (LES) with wall-adapting local-eddy viscosity (WALE) model and an all y+ wall function^13^. A polyhedral core mesh was combined with 18 prismatic layers at the walls (stretching parameter = 1.1, mean prismatic cell aspect ratio = 0.45–0.46), resulting in 10.5–13.3 M cells. Examining the percentage of resolved turbulent kinetic energy (TKE) in the dsAvalon at the highest ECMO flow rate (6 L/min) shows that the vast majority of energy containing structures are resolved on the grid (87% in the cannula and 92% for the RA). For the same cannula and flow rate, grid convergence was assessed on a coarse (2.13 M cells), medium (4.86 M cells) and fine (10.54 M cells) mesh. Time-averaged velocity (Fig. S1) and TAWSS (Figs. S2 and S3) in the cannula and right atrium showed acceptable convergence for the fine mesh which was used in all subsequent simulations. A non-Newtonian Quemada model was used for blood viscosity (Hematocrit of 30%)^14^. The venous vascular and atrium walls were assumed to be rigid based on imaging gated for atrial systole. The tricuspid valve was modelled to remain fully open. All simulations were run on the Tetralith (National Supercomputer Centre, Sweden) and Galileo Supercomputers (CINECA, Italy).

### Cannulation parameters

The dsAvalon and dsCrescent DLCs were first placed in a baseline position, with the reinfusion port in the middle of the RA (2 cm below the SVC junction) oriented toward the tricuspid valve, and run at ECMO flow rates of 2, 4 and 6 L/min. These ECMO flow rates represent the expected clinical operating conditions as well as a high flow rate scenario (6 L/min) to compare the cannulas under extreme conditions where significant ${R}_{f}$ is expected. It should be noted that this lies outside the operating range tested experimentally by the manufacturers. ${R}_{f}$, flow structures and IVC/SVC drainage fraction were analysed. Given the very similar haemodynamics, the dsAvalon design was used as a generic geometry to determine the effect of rotation and insertion depth on DLC ${R}_{f}$. Rotation was assessed at ± 30° and ± 60° relative to baseline position as this was considered a reasonable clinical range. Insertion depth was assessed at ± 4 cm relative to baseline, allowing assessment of the performance when the reinfusion port was positioned immediately above and below the RA.

### Hemodynamic metrics

${R}_{f}$ was calculated by assigning a passive scalar to the DLC infusion and determining the fraction of this scalar at the DLC drainage outlet. Wall shear stress (WSS), central to understanding hemolysis in medical devices and endothelial physiology, was assessed. Velocity streamlines were used to visualize flow structures. ${R}_{f}$, WSS and velocity results were time-averaged for 1 s (100 samples) to report the average hemodynamics. Caval pressures were evaluated by point probes, one placed at the mid IVC (6.5 cm below the cannula tip) and one placed at the mid SVC. To compute pressure differences from the cannula connectors to the RA, commonly referred to as the ‘pressure drop’, point probes were placed at the cannula inlet, outlet and central RA for both DLC designs. Pressures at the location of the real-world cannula connectors were extrapolated assuming a straight geometry, as our simulation domain began in the brachiocephalic veins. This allowed for direct comparison with experimental data. Pressure differences, calculated for the dsAvalon DLC using the patient averaged CFD model were compared with experimental results from Wang et al. using Ringer’s solution + 40% by volume red blood cells^15^ and the manufacturers data using water^16^. For comparison, a second order polynomial was fitted to each pressure difference dataset (mean R^2^ = 0.999) and mean differences were assessed over 2–6 L/min, these trendlines are plotted in the Supplementary material (Fig. S4).

### Ethics approval and consent to participate

All subjects gave informed consent and ethics approval was obtained from the Swedish Ethical Review Authority (Ethical permit 2018/438-31).

## Results

**Figure 2:**
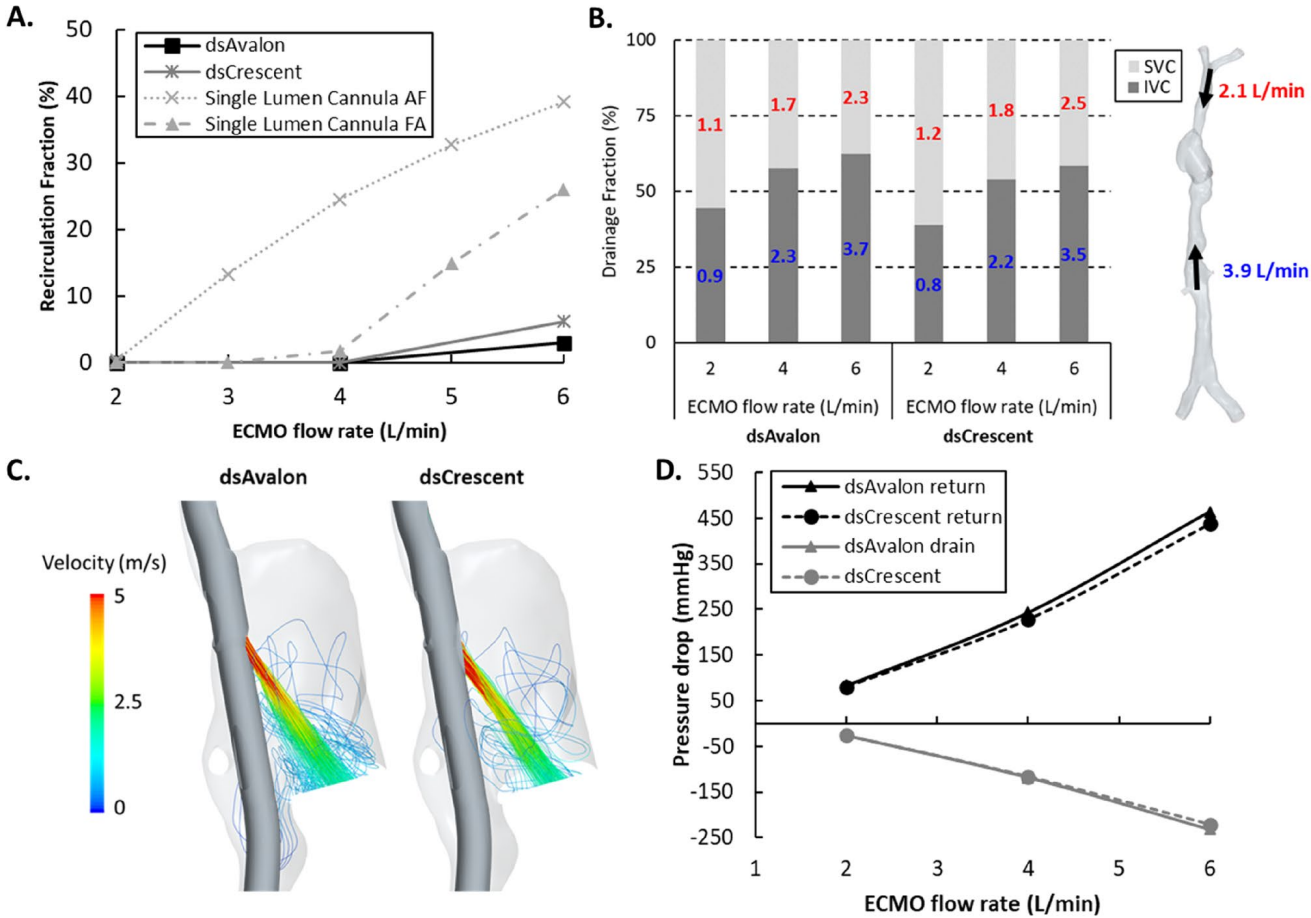
(**A**) Recirculation fraction (%) for the dsAvalon and dsCrescent dual-lumen cannula (DLC) as extracorporeal membrane oxygenation (ECMO) flow rate is increased. Single lumen cannula recirculation data^10^ in atrio-femoral (AF) and femoro-atrial (FA) configurations is also plotted for comparison. (**B**) *Left.* Drainage fraction (%) from the inferior vena cava (IVC) and superior vena cava (SVC) for both DLCs as ECMO flow rate is increased. The red and blue numbers denote drainage flow rates in L/min from the SVC and IVC, respectively. *Right*. Venous return flows applied to the model in L/min. (**C**) Time-averaged velocity streamlines exiting the reinfusion port from both DLCs at 4 L/min ECMO flow. (**D**) Return and drainage side pressure difference curves for both DLCs.

**Figure 3:**
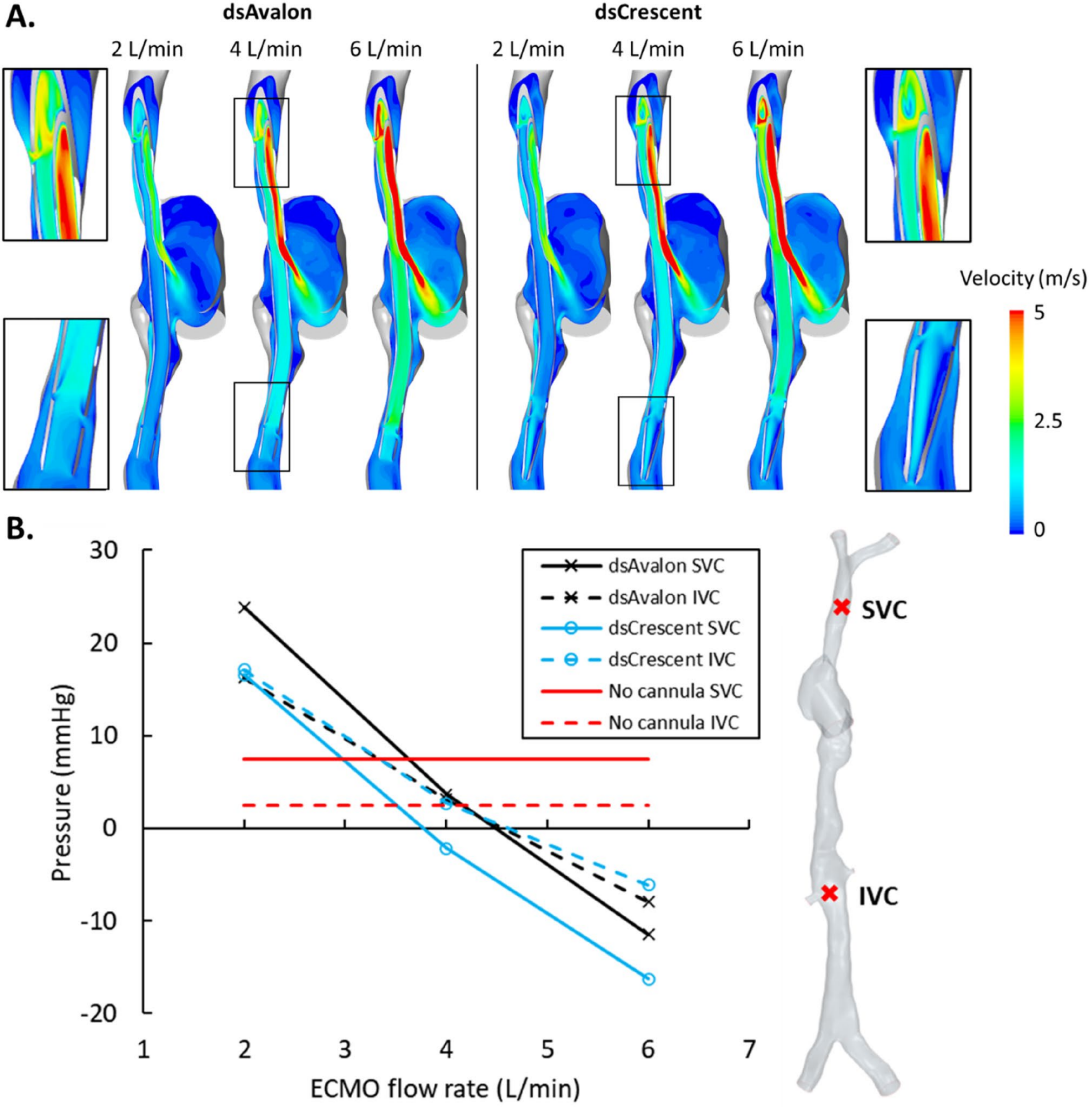
(**A**) Time-averaged velocities for the dsAvalon and dsCrescent dual-lumen cannulas (DLC) on a cross-sectional plane for all extracorporeal membrane oxygenation (ECMO) flow rates. (**B**) Caval pressures for both DLCs across all ECMO flow rates. These are compared with caval pressures when no cannulas are inserted. Point probe locations are indicated to the right.

In the baseline position, ${R}_{f}$ in both DLCs was low across all ECMO flow rates. Whilst being zero at 2 and 4 L/min, at 6 L/min ${R}_{f}$ increased slightly to 3.0–6.2%. Compared to data from SLCs in atrio-femoral and femoro-atrial configurations, using the same patient-averaged model^10^, DLC ${R}_{f}$ at the baseline position was much lower (Fig. 2A) with the exception of 2 L/min where both cannula strategies have near-zero recirculation. At 2 L/min, SVC drainage was favored by both DLCs. At higher ECMO flow rates both devices began to drain more blood from the IVC (Figs. 2B, 3A). For both DLCSs, caval pressures were abnormally high (16.2–23.9 mmHg) at low ECMO flow rates (2 L/min), dropping with increased flow, most noticeably in the SVC (Fig. 3B). The IVC/SVC drainage fractions at all flow rates were similar for both DLCs (mean difference ± SD = 4.3 ± 0.9%, Fig. 2B). Slight design differences in the reinfusion port design between the two cannula designs did not significantly affect return flow characteristics, both featuring a high velocity (> 5 m/s at 4 L/min), focussed jet (Fig. 2C). Both DLCs had nearly identical pressure drop curves (Fig. 2D).

**Figure 4:**
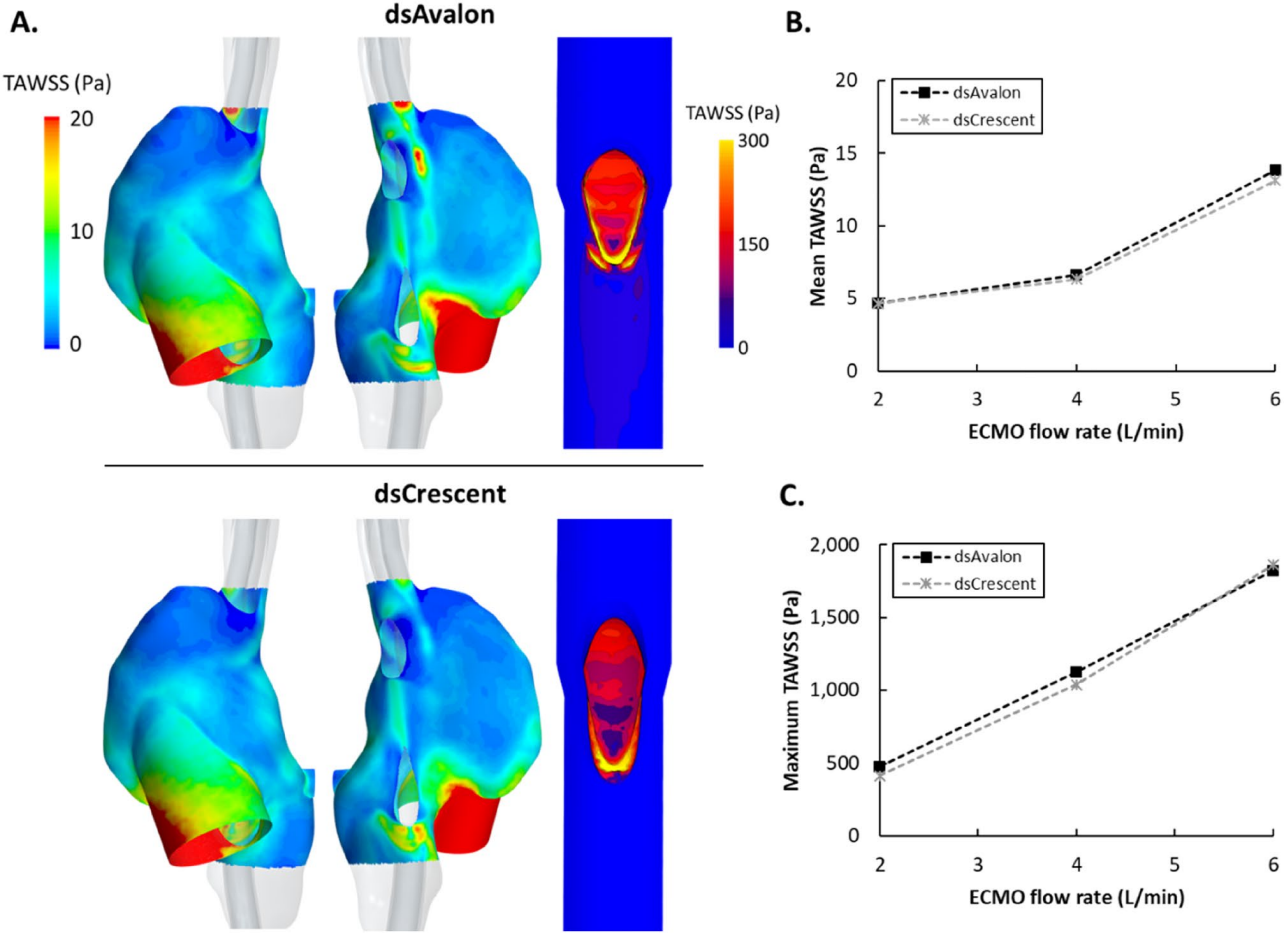
(**A**) Time-averaged wall shear stress (TAWSS) in the right atrium (left) and cannula (right) when using the dsAvalon (top) and dsCrescent (bottom) devices, at 4 L/min extracorporeal membrane oxygenation (ECMO) flow. (**B**) Mean TAWSS on the right atrium surface for both cannulas as ECMO flow rate is increased from 2 to 6 L/min. (**C**) Maximum TAWSS in both cannulas (occurring at the reinfusion port) as ECMO flow rate is increased from 2 to 6 L/min.

Surface average time-averaged WSS (TAWSS) at the atrial wall was similar for both DLCs and spatially consistent (Fig. 4A). As expected, surface averaged RA TAWSS increased with ECMO flow rate (Fig. 4B). Maximum RA TAWSS (Supplementary material Fig. S5) was higher for the dsAvalon at 2 L/min and higher for the dsCrescent cannula at 4 L/min (58.3 vs. 52.1 Pa) and 6/min (135.6 vs. 125.7 Pa). The reinfusion port design created a high TAWSS region at the distal edge of the reinfusion port (Fig. 4A). Maximum TAWSS was similar between the two devices (mean difference ± SD = 8.0 ± 4.9%), increasing with ECMO flow rate (Fig. 4C). Cannula surface average (internal and external) TAWSS (Supplemental material Fig. S6) was also similar in both DLCs (mean difference ± SD = 3.5 ± 0.5%).

**Figure 5:**
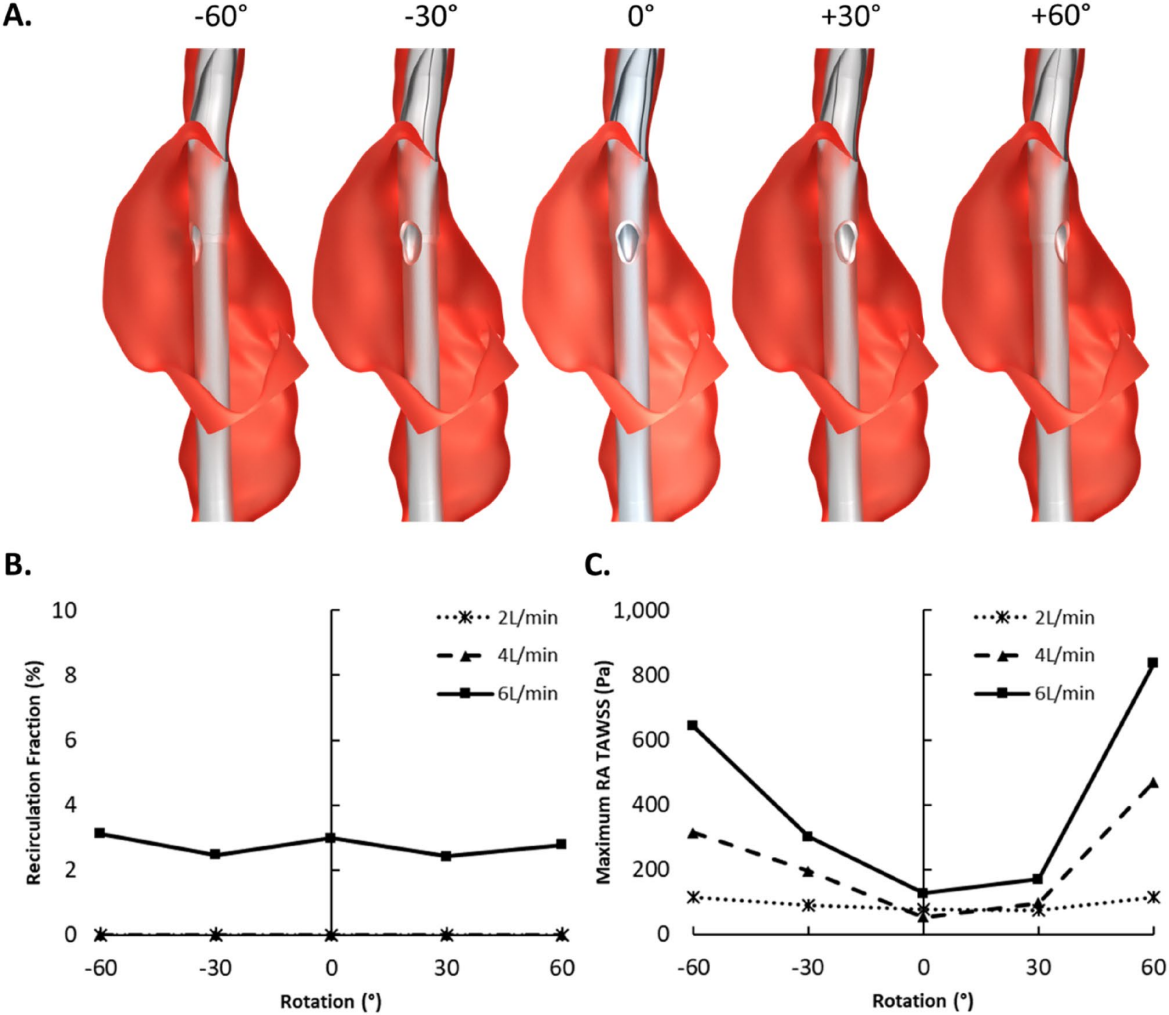
(**A)** Rotated bi-caval dual-lumen cannula (dsAvalon) geometries simulated. (**B**) Recirculation fraction for all rotations and extracorporeal membrane oxygenation (ECMO) flow rates. (**C**) Maximum time averaged wall shear stress (TAWSS) in the right atrium for all rotations and ECMO flow rates.

Rotation (Fig. 5A) of the bi-caval (bc) DLC (dsAvalon cannula) had little impact on ${R}_{f}$ (Fig. 5B). At 2 and 4 L/min the bcDLC retained its near zero ${R}_{f}$ throughout all rotations and at 6 L/min ${R}_{f}$ remained low (2.4–3.1%). However, RA TAWSS was impacted by cannula rotation (Fig. 5C) as maximum TAWSS was high for extreme rotations and minimal at, or close to, baseline position. Mean RA TAWSS (Supplemental material Fig. S7) was higher when the cannula was rotated towards the patients right (negative direction).

**Figure 6:**
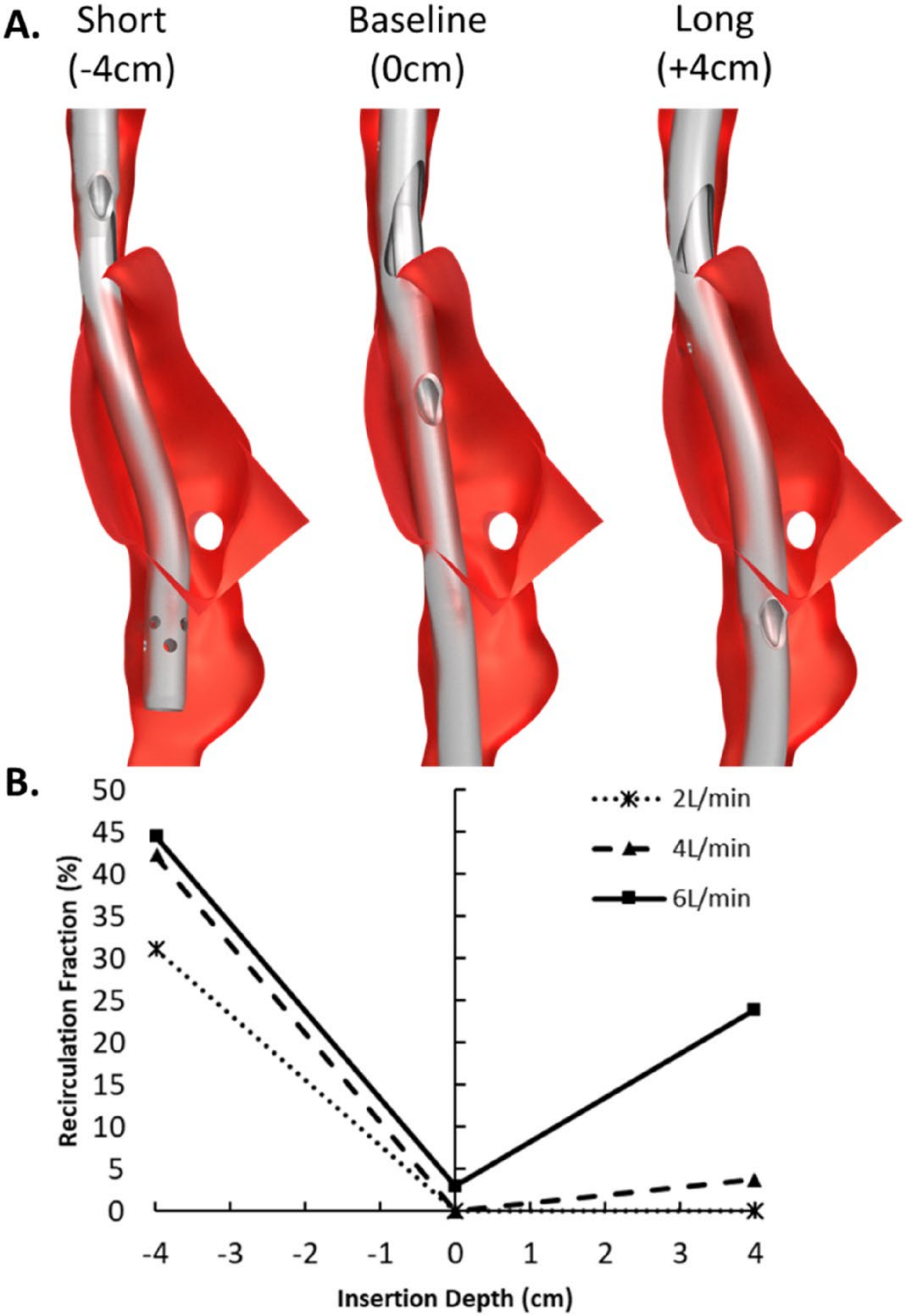
(**A**) Bi-caval dual-lumen cannula (dsAvalon) geometries with different insertion lengths. (**B**) Recirculation fraction (%) for all insertion depths and extracorporeal membrane oxygenation flow rates.

Cannula insertion depth (Fig. 6) had a significant impact on ${R}_{f}$ at all ECMO flow rates. Short insertion increased ${R}_{f}$ (31.2–44.6%). Long insertion depths caused high ${R}_{f}$ at 6 L/min (24.0%) and a relatively small increase at 4 L/min (3.8%). At 2 L/min a near-zero ${R}_{f}$ was retained even with a long insertion depth.

**Figure 7:**
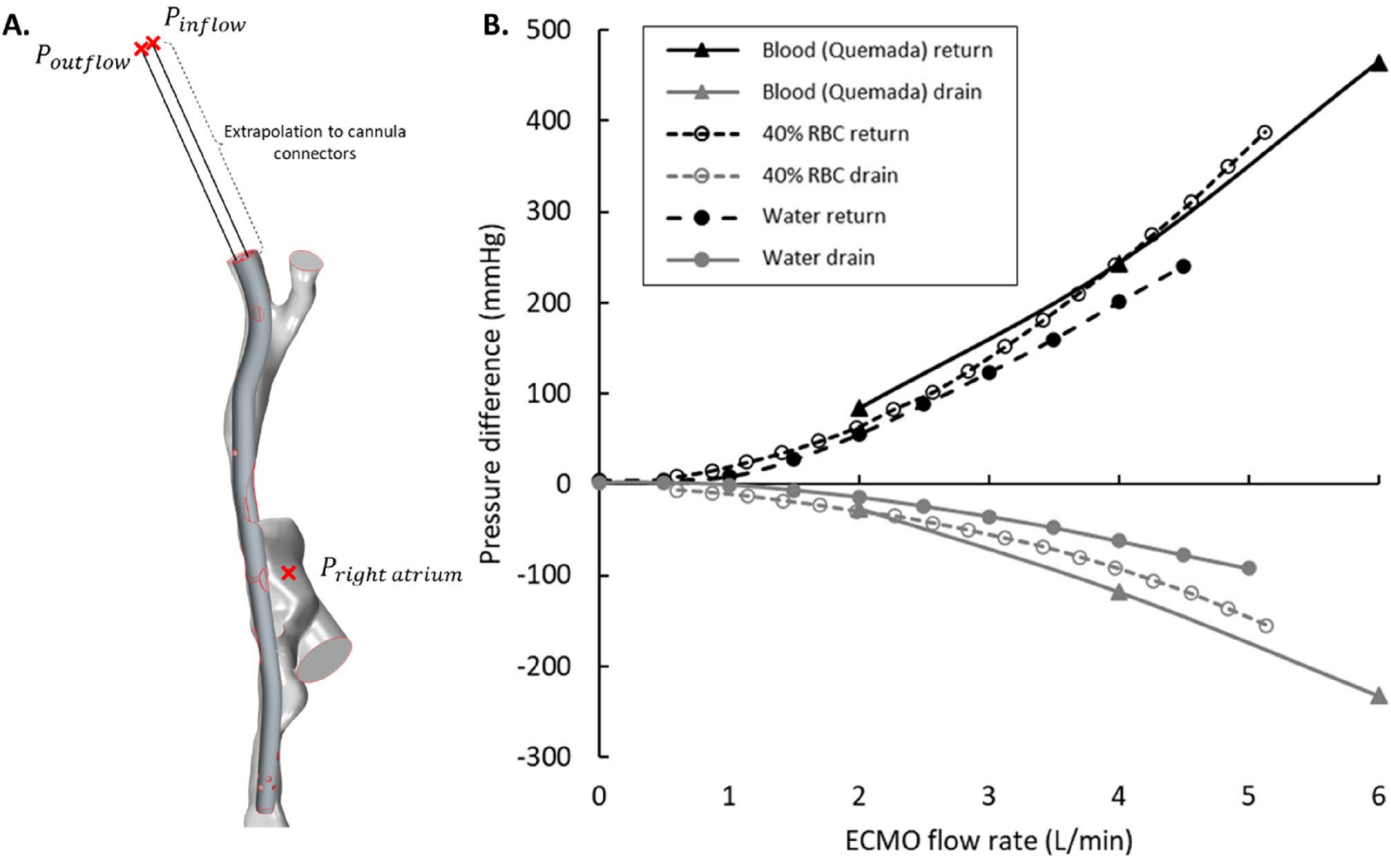
(**A**) Schematic of pressure difference ($${P}_{inflow/outflow}-{P}_{right atrium})$$ calculation in the model. (**B**) Pressure difference curves on the return and drainage side of the 27Fr Avalon Elite dual-lumen cannula as measured in experimental data from Wang et al. using Ringer’s solution with 40% red blood cells (40% RBC)^15^ and Maquet data sheet using water^16^ compared to those calculated by our dsAvalon model (Blood).

Comparing the dsAvalon pressure drops from the CFD model with experimental results showed reasonable agreement (Fig. 7). The pressure drop curves calculated from the CFD model were closer to experimental results obtained using a RBC suspension (return and drainage mean difference ± SD = 20.9 ± 16.3 mmHg and 20.0 ± 8.5 mmHg, respectively) than the manufacturer data based on water (return and drainage mean difference ± SD = 41.0 ± 5.0 mmHg and 56.4 ± 24.8 mmHg, respectively).

## Discussion

In this study, the performance of two generic designs based on the Maquet Avalon and MC3 Crescent DLC were compared via two models both downscaled to 27Fr. The two devices performed similarly, characterized by low ${\mathrm{R}}_{\mathrm{f}}$ and comparable IVC/SVC drainage fractions across all ECMO flow rates. Both designs produced a high velocity jet of return flow resulting in elevated cannula and RA TAWSS. Caval pressures were abnormally high when simulating either DLC at a low ECMO flow rate. Pressure drop curves for both devices were nearly identical. Comparing pressure difference data from the CFD model for the dsAvalon DLC with experimental data in literature shows reasonable agreement. Assessing the impact of malpositioning on the bcDLC (dsAvalon) showed that rotation had minimal effect on ${R}_{f}$ whilst insertion depth had a strong relationship. Long insertion depth increased ${R}_{f}$ at high ECMO flow rates whilst short insertion raised ${R}_{f}$ for all flow rates.

### Performance of the dsAvalon and dsCrescent DLCs

Despite several design differences the hemodynamics of the two devices are similar. Under ideal placement, both devices achieve near zero ${R}_{f}$ at low and moderate ECMO flow rates. ${R}_{f}$ remained low (< 7%) even at high ECMO flow. Compared to SLCs placed in the same computational model (Fig. 2A)^10^, the ${R}_{f}$ of DLCs are considerably lower at flows > 4 L/min (> 2 L/min for atrio-femoral configuration). The CFD model here clearly shows the superior ${R}_{f}$ performance of DLCs, previously lacking^5^.

The CFD model also provides hemodynamic data to elucidate the reason for the superior ${R}_{f}$ performance of DLCs. ${R}_{f}$ in VV ECMO increasingly appears to be coupled with the availability of return venous flow at drainage sites (Fig. 2B)^10,17^. A critical advantage of DLC design is the dual drainage sites in the SVC and IVC meaning that there is a greater supply of venous return flow to drain. The ratio of flow drained from the SVC and IVC adapts as ECMO flow rates increase (Fig. 2B). At low ECMO flow rates the DLCs favor draining SVC blood which is expected as the holes are closer to the suction source, reflecting results reported for a pediatric DLC^18^ and in adult ECMO SLCs^19,20^. As ECMO flow rate is increased, SVC blood supply (35% of total, 2.1 L/min) is low compared to the increased total drainage flow rate creating a negative pressure (Fig. 3B). Consequently, IVC drainage is favored due to a higher-pressure gradient between the cannula drainage holes and the available venous return flow from the lower body and visceral organs. In SLC cannulation, when the drainage rate exceeds the venous supply (of the one vena cava), vena cava pressure drops, increasing the likelihood of chattering and more oxygenated blood will be drawn from the RA, i.e. recirculation occurs.

Both devices impacted caval pressures greatly when compared with the cannula-free equivalent (Fig. 3B). Vena cava pressures were abnormally high at 2 L/min ECMO flow (Fig. 3B). These high pressures were not observed under single lumen cannulation (Supplemental material, Fig. S8) in the same computational model^21^. This is likely due to the obstruction that the DLC causes within the venae cavae. This was particularly pronounced at the SVC-RA junction. At low ECMO flow rates, relatively limited venous return flow was drained (Fig. 2B). This flow was also partially obstructed from entering the RA, increasing vena cava pressure (Fig. 3B). The effect of this obstruction was negated at high ECMO flow rates where a high proportion of venous return flow was drained and a negative caval pressure developed. Though a negative caval pressure brings with it the risk of chattering and increased ${R}_{f}$. Whilst larger diameter DLC cannulas may be selected for their lower pressure drops, the obstruction to venous flow observed in the present study is likely to worsen. This finding is consistent with results from the ELSO Registry showing increased odds ratio for intracranial hemorrhage of 2.74 (95% CI 1.06–7.09, p = 0.03) comparing the use of 27Fr versus 31Fr bcDLC^22^. Though the direct link is yet to be established, these results further support our previous findings^21^ that ECMO cannula performance is highly dependent on the balance between ECMO drainage flow and venous return flow in each vena cava.

### Reinfusion port design

The highly focussed reinfusion port in both DLCs is designed to direct return blood immediately through the tricuspid valve, reducing the blood flow fraction that could migrate to the two drainage locations. This feature creates localised regions of very high shear stress at the lip of the cannula port (> 413 Pa for all flow rates) (Fig. 4A,C). The nature of all artificial circuit components is that they expose blood to high shear stresses. Physiological shear stresses are generally low (< 10 Pa)^23,24^, in part due to lower flow rate/vessel diameter ratios, compliant walls, and unique morphology. Non-physiological shear stresses (NPSS) however affect the carefully managed balance of coagulability in the ECMO patient, prone to both bleeding and thrombotic events. The physiological implications of NPSS are myriad including hemolysis, von Willebrand factor (vWf) fragmentation and platelet activation, adhesion, and apoptosis^23^. Consequently, NPSS is a key design criterion for ECMO pumps. The results from this study show that DLCs cause NPSS equivalent to those in ECMO pumps^25^, and consideration of lowering WSS might improve DLC hemocompatibility. Furthermore, the focussed jet of blood leaving the reinfusion port creates high WSS in the RA itself (Fig. 4B, S5), potentially further increasing thrombogenicity.

### DLC rotation

Current DLC design is based on targeted delivery of oxygenated blood through the tricuspid valve. Consequently, correct angular orientation of the DLC becomes a primary objective in cannulation. Literature is scarce on the impact of DLC rotation. Jamil et al. have assessed the impact of rotation using a 13Fr Origen (OriGen Biomedical, Austin, Texas, USA) neonatal DLC (now discontinued) in a computational model of pediatric ECMO^18^. This device seemingly had high ${R}_{f}$ (38%) even with correct position, likely due to the very close proximity of the reinfusion and drainage ports, by design being a cavo-atrial DLC. With rotation of the device ~  ± 60° (estimated via figures in Ref.^18^), ${R}_{f}$ ranged from 32 to 39%, ultimately having little impact. Similarly, the results from the present study suggest that rotation of the cannula i.e. changing direction of the reinfusion port had no significant impact on ${R}_{f}$ (Fig. 5B). This finding challenges the widespread belief that DLCs reduce ${R}_{f}$ by directing return flow to the tricuspid valve. Thus, we question the value of such a highly focussed reinfusion port given the high shear rates the blood is exposed to. These results may, hypothetically, inform iterative redesign of the reinfusion port/zone for increased biocompatibility.

### Insertion depth

Data on the impact of DLC insertion depth is similarly deficient. Körver et al. report an ultrasound dilution technique for quantification of ${R}_{f}$ in the Avalon DLC, presenting three cases^7^. One case, where a 27Fr device was inserted in too far to near the hepatic vein, shows ${R}_{f}$ of 45% at 4.7 L/min ECMO flow. These results are consistent with the increased ${R}_{f}$ we observe with deeper insertion at higher ECMO flow rates (Fig. 6B). The cannula malposition in this case is likely more severe than that presented in our study, with the cannula being inserted deeper and rotated away from optimal orientation. The most novel finding from the present study is the very sharp increase in ${R}_{f}$ for all ECMO flow rates when insertion depth is too short, a clinical observation also experienced by the authors. A potential explanation for this is the relative lack of venous return flow in the SVC. When the reinfusion port sits in the SVC, the proximal drainage holes create a lower pressure in the SVC, drawing a high proportion of the return flow away from the RA and back into the ECMO circuit. The natural funnel shape of the SVC-RA junction and the obstruction of the relatively large diameter DLC at the narrow opening further contribute to this effect. The direction of the reinfusion jet, again, is less critical to ${R}_{f}$.

### Pressure drop quantification

We see reasonable agreement between previous experimental data and CFD results for the 27Fr dsAvalon. Our CFD results show better agreement with Wang et al.’s results obtained with Ringer’s solution + 40% RBCs than the manufacturers data based on experiments with water, underestimating the pressure drop by a mean of 16% and 46% for return and drainage sides, respectively. These observations support the findings of Broman et al. that flow mediums can affect pressure drop curves in the DLC^26^. This further supports the use of a standardised CFD approach for testing cannula performance where hematocrit, venous inflows (renal, iliac and brachiocephalic), positioning and morphology can be controlled.

### Model limitations

The CFD model applied here has several limitations. Firstly, the patient-averaged model is based on a small number of healthy volunteers, geometry may differ significantly from the typical ECMO patient. Secondly, the walls of the model are assumed to be rigid. The unknown wall thickness, material properties and external constraints of vessels mean that a fluid structure interaction (FSI) model would require several more assumptions and likely not improve accuracy. Whilst such an approach would increase the computational cost of the simulations significantly. Thirdly, inflows to the model were assumed to be constant, this may reflect more closely the deeply sedated rather than awake ECMO patient. Constant inflows have been assessed previously to have little effect on time-averaged velocities in the RA^8^. Realistic transient inlet waveforms for an ECMO patient were not available. Were these to be quantified and added to the model they may have had some impact on caval TAWSS as pulsatility tends to flush out stagnant zones. In the present study, TAWSS in the cannula and RA arising from the constant cannula return flow was of primary interest. Fourthly, after establishing the IVC/SVC flow split, flows were distributed by inlet area. Patient-specific measurement of these inflows was not available. Despite these simplifications, previous comparison with clinical data suggests that the CFD model accurately describes ${R}_{f}$ in the average ECMO patient^10^. The fifth limitation relates to the 3D DLC models. Using available samples, the 3D models simulated were downscaled versions of slightly larger cannulas (30Fr Crescent and 31Fr Avalon) whilst cannula wall thicknesses were kept constant. Additionally, there is no 27Fr Crescent cannula in the current range, though one was created to allow for a fair hemodynamic comparison between the two designs. Comparison of the pressure drop curve from the dsAvalon cannula shows reasonable agreement with experimental data indicating that the geometry was reproduced accurately. Further to this, only the dsAvalon was assessed at a range of rotations and insertion depths, justified by the similarity of the two devices. Lastly, the cannula insertions follow the vessel centerlines, minimising the cannula/venous wall contact. This may impact drainage through some side-holes where wall contact might normally impede flow. Simulating clinical insertion with a finite element analysis (FEA) model in the future may yield more accurate cannula positioning. Similarly, imaging cannulated ECMO patients could provide this data, though this presents significant practical obstacles.

## Conclusions

The two generic bi-caval DLC designs performed similarly. Both offered lower recirculation compared to dual site cannulation, especially in the higher flow ranges. Like SLCs, DLC performance is highly dependent on the relationship between vena cava supply and drainage rates. DLC insertion depth was critical where both too shallow and too deep insertion caused high recirculation at different flow rates. The focussed reinfusion ports created very high shear rates, but rotation per se did not affect recirculation with correct insertion depth. These data challenge the value of the current reinfusion port design and our understanding of why DLCs have superior clinical performance.

## Supplementary Information

Supplementary Information.

## Data Availability

The datasets used and/or analysed during the current study are available from the corresponding author on reasonable request.
