# Supplementary material for: Hemodynamic and recirculation performance of dual lumen cannulas for venovenous extracorporeal membrane oxygenation
Source: Sci Rep. 2023 May 8;13:7472. doi: 10.1038/s41598-023-34655-1 (PMC10167322; doi:10.1038/s41598-023-34655-1)
Supplement: Supplementary file 1 — Supplementary Information. [file 41598_2023_34655_MOESM1_ESM.docx]

Supplementary Material

**Hemodynamic and Recirculation Performance of Dual Lumen Cannulas for Venovenous Extracorporeal Membrane Oxygenation**

Louis P. Parker PhD¹, Anders Svensson Marcial PhD² ³, Torkel B. Brismar MD PhD² ³, Lars Mikael Broman MD PhD⁴ ⁵, Lisa Prahl Wittberg PhD¹.

1. FLOW, Department of Engineering Mechanics, Royal Institute of Technology, KTH, Stockholm, Sweden.

2. Department of Clinical Science, Intervention and Technology at Karolinska Institutet, Division of Medical Imaging and Technology, Stockholm, Sweden.

3. Department of Radiology, Karolinska University Hospital and Karolinska Institutet, Stockholm, Sweden. ECMO Centre Karolinska, Pediatric Perioperative Medicine and Intensive Care, Karolinska University Hospital, Stockholm, Sweden.

4. ECMO Centre Karolinska, Pediatric Perioperative Medicine and Intensive Care, Karolinska University Hospital, Stockholm, Sweden.

5. Department of Physiology and Pharmacology, Karolinska Institutet, Stockholm, Sweden.

**Corresponding Author:** Professor Lisa Prahl Wittberg

**Email:** prahl@kth.se **Phone:** +46 73-675 64 64

**Address:** Osquars backe 18, SE-100 44 Stockholm, Sweden.

This supplementary material contains several figures which add context to the main text. As in the main text, to delineate that these are not direct reconstructions of the off-the-shelf products but downscaled (ds) representations we refer to the cannulas as dsAvalon and dsCrescent. Figures S1, S2 and S3 describe the grid convergence study conducted on the dsAvalon cannula at 6L/min ECMO flow rate. To compare pressure curves derived from computational fluid dynamics (CFD) simulations and experimental data we fitted second-order polynomials to all data sets. This allowed for comparison of data collected across different intervals and flow rate ranges. These trendlines are shown graphically in Fig S4. In the manuscript several mean and maximum time-averaged wall shear stress (TAWSS) graphs are presented for the right atrium (RA) and cannulas. For completeness, the maximum RA TAWSS (Fig. S5) and mean cannula TAWSS (Fig. S6) plots not presented in the main text are included. Similarly, the maximum RA TAWSS from the dsAvalon cannula under rotation is presented in the main text. We have therefore included the corresponding mean RA TAWSS plot here (Fig. S7). Finally, we discuss in the manuscript the impact of the dual-lumen cannula (DLC) on caval pressures at low ECMO flow rates. To show that this is unique to DLCs we have extracted caval pressure data from a previous study using single lumen cannulas (Fig. S8).


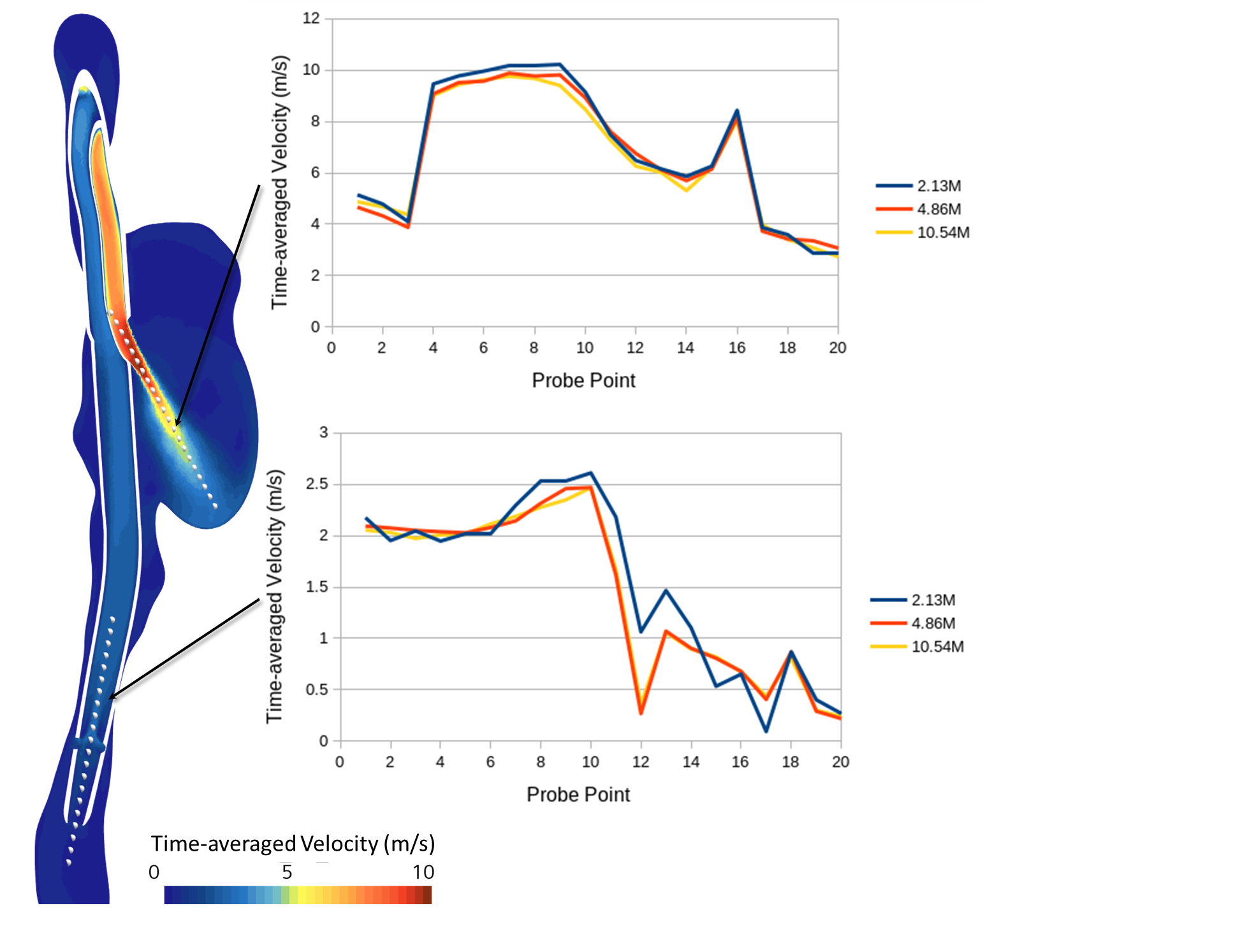


Figure S1. Time-averaged velocity grid convergence results obtained for the dsAvalon dual lumen cannula. ECMO flow was 6L/min and three mesh densities were assessed: coarse (2.13M cells), medium (4.86M cells) and fine (10.54M cells). All subsequent simulation were run on the fine mesh. Results are plotted for two line probes at the cannula reinfusion port (top) and cannula tip (bottom).


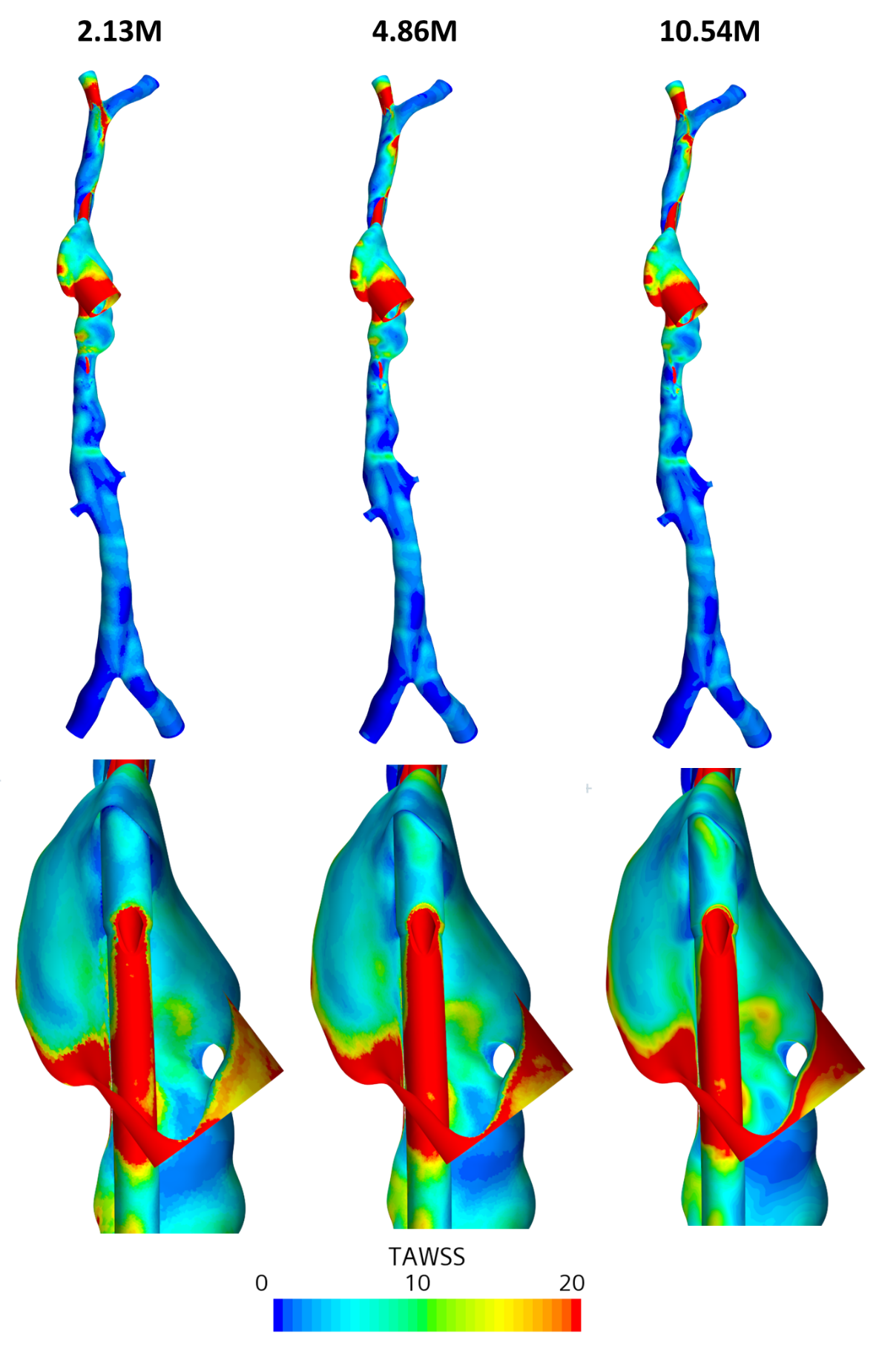
Figure S2. Time-averaged wall shear stress (TAWSS) for the coarse, medium and fine meshes shown at the venae cavae walls (top) and cannula reinfusion port (bottom).


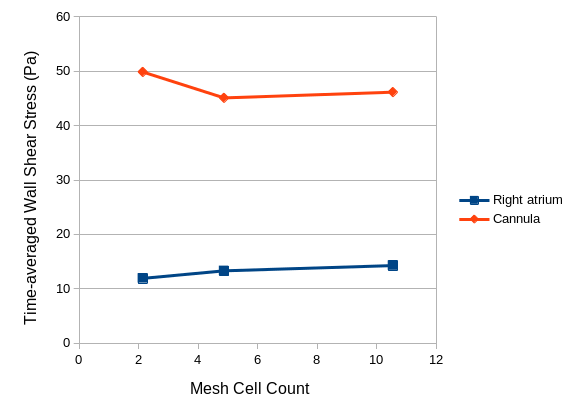
Figure S3. Surface averages of time-averaged wall shear stress (TAWSS) in the right atrium and cannula wall, plotted the coarse, medium and fine meshes.


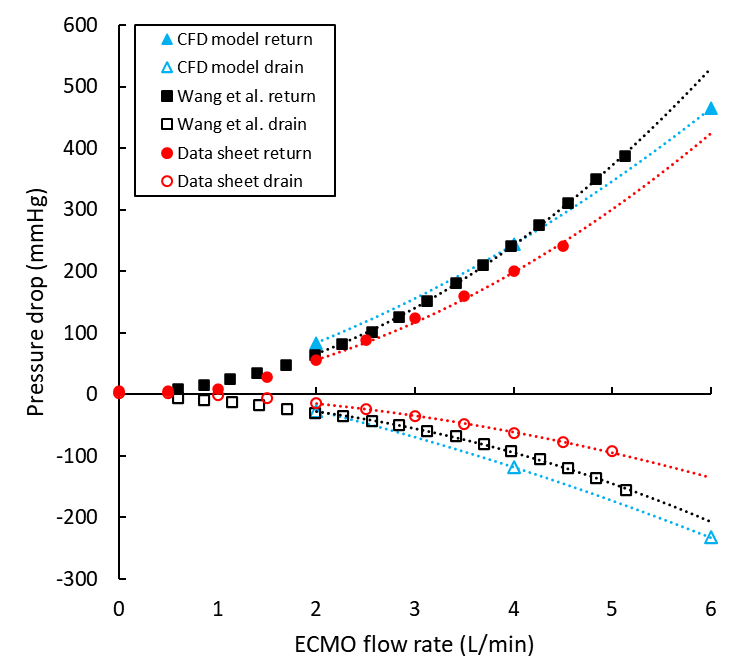


Figure S4. Second order-polynomial trendlines fitted to computational fluid dynamics (CFD) and experimental pressure difference data for the down-scaled (ds)Avalon bi-caval dual-lumen cannula compared to experimental data from Wang et al. using Ringer’s solution with 40% red blood cells^1^, and Maquet data sheet using water^2^. The cannula is referred to as downscaled as a 31Fr Avalon cannula sample was measured, reconstructed and scaled to 27Fr for insertion into the patient-averaged model.


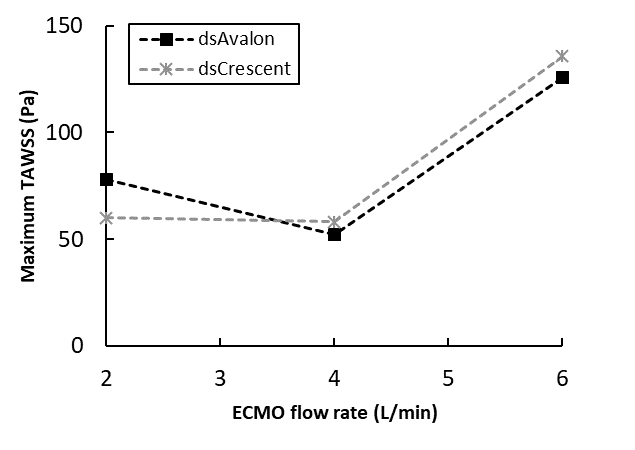
Figure S5. Maximum right atrium time-averaged wall shear stress (TAWSS) for the downscaled (ds)Avalon and dsCrescent dual-lumen cannulas. The cannulas are referred to as downscaled as 31Fr (Avalon) and 30Fr (Crescent) cannula samples were measured, reconstructed and scaled to 27Fr for insertion into the patient-averaged model.


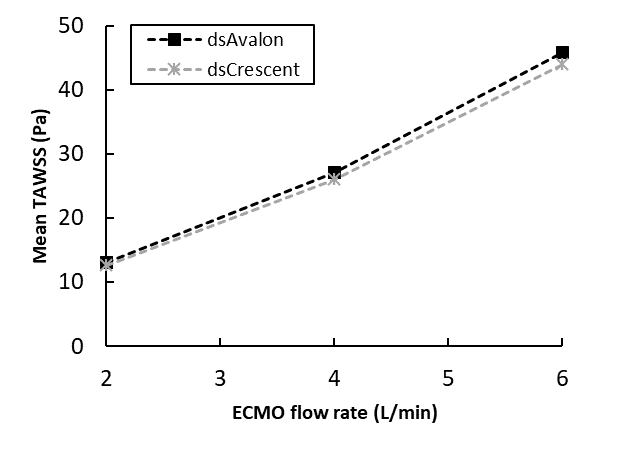
Figure S6. Mean cannula time-averaged wall shear stress (TAWSS) for the downscaled (ds)Avalon and dsCrescent dual-lumen cannulas. The cannulas are referred to as downscaled as 31Fr (Avalon) and 30Fr (Crescent) cannula samples were measured, reconstructed and scaled to 27Fr for insertion into the patient-averaged model.


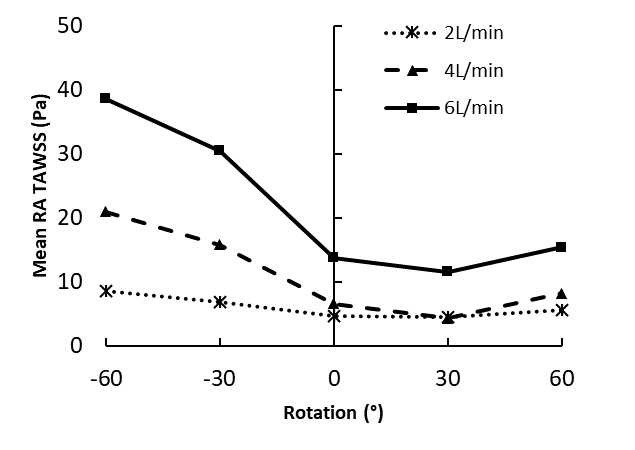
Figure S7. Mean right atrium time-averaged wall shear stress (TAWSS) for the downscaled (ds)Avalon cannula when rotated from -60 to +60°. The cannula is referred to as downscaled as a 31Fr Avalon cannula sample was measured, reconstructed and scaled to 27Fr for insertion into the patient-averaged model.


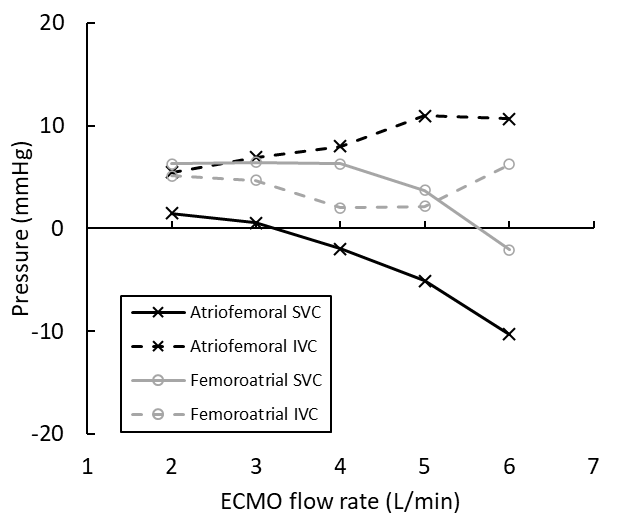
Figure S8. Vena cava pressure data under atrio-femoral and femoro-atrial single-lumen cannula ECMO from a previous study.^3^

**References**

1 Wang, S. *et al.* Hemodynamic Evaluation of Avalon Elite Bi-Caval Dual Lumen Cannulas and Femoral Arterial Cannulas. *Artificial Organs* **43**, 41-53, doi:<https://doi.org/10.1111/aor.13318> (2019).

2 Maquet Cardiopulmonary GmbH. (ed Maquet Cardiopulmonary GmbH) 1-4 (Maquet Cardiopulmonary GmbH, Rastatt, Germany, 2021).

3 Parker, L. P., Marcial, A. S., Brismar, T. B., Broman, L. M. & Prahl Wittberg, L. Cannulation configuration and recirculation in venovenous extracorporeal membrane oxygenation. *Sci Rep* **12**, 16379, doi:10.1038/s41598-022-20690-x (2022).
